# Wernicke Encephalopathy in a Patient With Bipolar Disorder: A Case Report

**DOI:** 10.7759/cureus.40646

**Published:** 2023-06-19

**Authors:** Kateryna Chepenko, Muhammad Humayoun Rashid, Ulviyya Turabova, Tigran Kakhktsyan, Sindhu Chadalawada, Ali Abdulsahib, Aliaa Mousa, Shafaq Bokhari

**Affiliations:** 1 Internal Medicine, Capital Health Regional Medical Center, Trenton, USA

**Keywords:** bilateral ophthalmoplegia, alcohol use encephalopathy, bipolar disorders, non-alcoholic wernicke's encephalopathy, wernicke encephalopathy

## Abstract

Wernicke encephalopathy (WE) is a combination of neurological findings including confusion, ataxia, and ophthalmoplegia. It is most commonly associated with patients who have a history of alcohol abuse. This aspect leads to the majority of cases going undiagnosed in non-alcoholic patients who have other potential thiamine deficiency-causing conditions such as malignancy, chronic kidney disease (CKD) on hemodialysis, hyperemesis gravidarum, and psychiatric disorders leading to starvation and malnourishment. Here we present the case of a 59-year-old female patient with decompensated bipolar disorder who came in with altered mental status and multiple syncopal episodes. On examination, she was completely confused and had a fixed gaze. She was worked up for broad differential diagnoses including stroke, arrhythmias, seizures, drug intoxication, and infections. But due to her severely malnourished appearance, Wernicke’s encephalopathy was suspected early on, and she was started on thiamine therapy, to which she responded well. It was also confirmed by an MRI of the brain showing flair in the bilateral medial thalamic region. Therefore, to suspect the presence of WE in non-alcoholic patients with psychiatric disorders and to differentiate behavioral symptoms from delirium and encephalopathy is difficult and requires a high degree of clinical suspicion.

## Introduction

Wernicke’s encephalopathy (WE) is a neurological condition that is associated with a deficiency of vitamin B1 (thiamine). It is usually characterized by the presence of mental confusion, gait ataxia, and ophthalmoplegia, characterized by conjugate gaze palsies including nystagmus [1]. It is most commonly associated with alcohol abuse; therefore, clinical suspicion is higher in patients presenting with increased alcohol intake and altered mental status. However, this bias can lead to WE going undiagnosed in many non-alcoholic patients, and it can progress to Korsakoff syndrome, which is irreversible and has a very high mortality rate [2]. Studies have shown it to be prevalent in non-alcoholic patients as well [3, 4]. In a study based on autopsy specimens, the prevalence of WE in non-alcoholics ranged from 0.8% to 2.8% [3].

Multiple case reports have been published on the association of Wernicke’s encephalopathy with conditions such as hyperemesis gravidarum, post-gastrointestinal surgery, malignancy, chronic kidney disease (CKD) on hemodialysis, and anorexia nervosa [5, 6]. The reason behind all of these is the same: thiamine deficiency. Here we encountered a case of a 59-year-old woman with a past medical history of asthma and bipolar disorder who came in with altered mental status and severe cachexia and was found to have full-blown Wernicke's encephalopathy.

## Case presentation

We present the case of a 59-year-old female patient with a medical history of asthma, hyperlipidemia, and bipolar disorder. The patient was admitted to the hospital due to a syncope episode at home and mental changes, including disorientation, confusion, and malnutrition. According to information obtained from the family, the patient had been living independently and maintaining employment. However, over the past few months, she had been exhibiting confusion and deviations from her baseline behavior. She failed to answer phone calls and appeared confused during conversations. Concerned about her well-being, the family visited her residence and discovered expired food in the fridge. Additionally, they noticed a significant weight loss since their last encounter, and the patient complained of not sleeping a couple of days in a row.

Upon physical examination, the patient presented with a weight of 60 kg, no documented previous baseline weight, a BMI of 23, a temperature of 36.7°C, a blood pressure of 127/73 mmHg, and a pulse rate of 88/min. She demonstrated difficulty answering questions and provided repetitive answers to different queries. The patient displayed an inability to follow simple commands and impaired visual tracking. Notably, her left pupil was fixed with no light reflex, while her right pupil exhibited reactivity to light. Increased muscular tone and hyperreflexia were observed in her extremities. Apart from these findings, the remainder of the neurologic examination was either unremarkable or incomplete. The patient appeared to stare at the wall. There was no documentation of medication for the treatment of bipolar disorder, and no recent prescription refills were found at the pharmacy.

Initial laboratory test results, including sodium, creatinine, glucose, amylase, lipase, and blood coagulation parameters, fell within normal ranges, but she was found to have electrolyte abnormalities such as mild hypokalemia, hypomagnesemia, low protein, and low albumin (Table 1).

**Table 1:** Laboratory findings of the patient on admission

	Patient's findings	Reference range
White blood cells	10.5 x10*3/mcL	4-10
Hemoglobin	10.3 g/dL	11.2-15.7
Platelet count	305X10^3/mcL	150-400
Sodium level	145 mmol/L	137-145
Potassium level	3.1 mmol/L	3.5-5.1
Chloride level	110 mmol/L	98-107
Carbon dioxide (CO2)	23 mmol/L	22-30
Blood urea nitrogen (BUN)	5 mg/dL	7-17
Creatinine level	0.41 mg/dL	0.52-1.04
Protein total	5.4 g/dL	6.5-8.5
Albumin	2.7 g/dL	3.5-5.0
Magnesium	1.3 mg/dL	1.6-2.3
Thyroid stimulating hormone (TSH)	1.288 mclntUnit/mL	0.465-4.680

**Figure 1:**
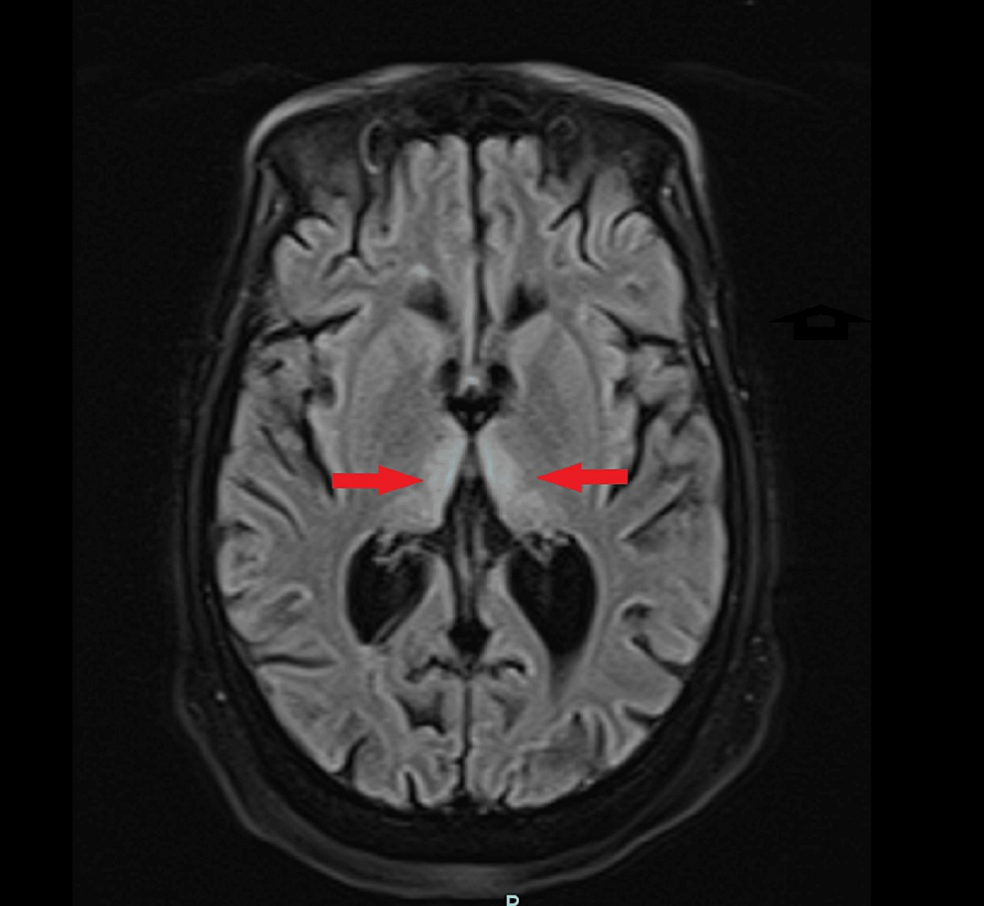
On a magnetic resonance imaging (MRI) scan of the brain, red arrows show a symmetric hypertense signal within the bilateral medial thalami region.

**Figure 2:**
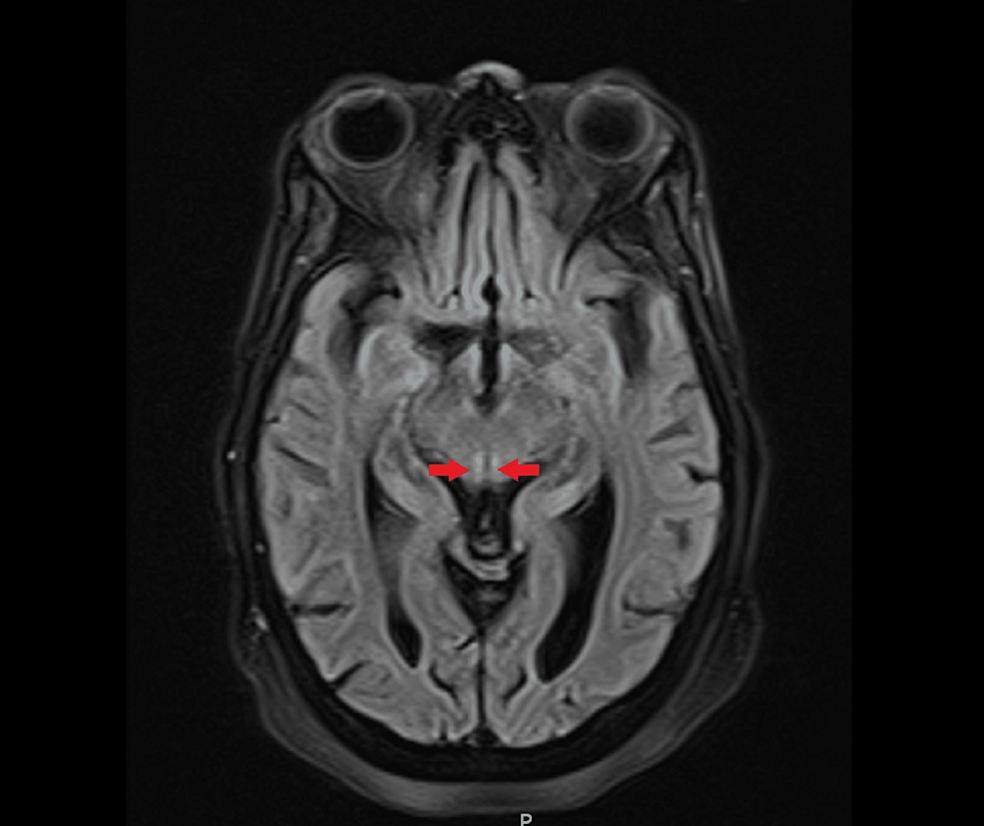
On magnetic resonance imaging (MRI) of the brain, the red arrows show a symmetric hypertense signal within the periaqueductal gray matter.

No seizures or epileptiform discharges were seen in the electroencephalogram (EEG). Given the broad range of possible diagnoses, consultations were sought from the neurology and psychiatry departments. The CT scan of the head showed advanced volume loss and microvascular ischemia. Subsequently, an MRI of the brain was performed, revealing symmetric fluid-attenuated inversion recovery (FLAIR) and diffusion-weighted MRI (DWI) hyperintense signals within the bilateral medial thalami and along the periaqueductal gray matter, as shown in Figures 1-2, respectively. These findings were suggestive of possible sequelae of Wernicke's encephalopathy.

In light of these findings, the patient was initiated on intravenous thiamine 500 mg three times daily for three days, followed by a reduced dosage of 250 mg intravenous thiamine for an additional three days. After just one day of treatment, the patient's confusion improved, and she became more alert and oriented to the place and herself. She recognized her family members and asked appropriate questions. Intravenous thiamine administration was completed, and the patient was transitioned to oral medication. Subsequently, she was discharged from the hospital on the same day.

## Discussion

The patient that we encountered here presented with acute delirium, multiple syncopal episodes, and ophthalmoplegia. On physical examination, the patient was found to be dehydrated and severely malnourished. Syncopal episodes were thought to be due to dehydration and generalized weakness because no arrhythmias were noticed and orthostatic vitals were positive. A broad differential diagnosis for acute delirium was made, including drug intoxication, infectious etiologies like urinary tract infection (UTI) or meningoencephalitis, seizure activity leading to a postictal state, or vitamin deficiency leading to Wernicke’s encephalopathy. The urine toxicology screen, blood ethanol level, acetaminophen level, and salicylate level were normal, ruling out major drug intoxications. The white blood count was normal, and the urinalysis and chest x-ray were unremarkable, ruling out pneumonia and UTI. An electroencephalogram ruled out any epileptiform changes. A CT scan of the head was done, which showed advanced volume loss without any intracranial hemorrhage or mass effect. The neurologist decided against doing a lumber puncture because there were no clinical signs suggesting meningoencephalitis. The diagnosis was narrowed down to Wernicke's encephalopathy in the setting of confusion and ophthalmoplegia. Ataxia and other motor examinations were difficult to assess because of severe generalized weakness and ambulatory dysfunction. The patient was able to move all four limbs in the bed. An MRI of the brain was ordered, which showed T2-flair hyperintense foci within the bilateral medial thalami and along the periaqueductal gray matter, which was pathognomic for WE [7]. The patient was started on the recommended dose of intravascular thiamine therapy, which is 500 mg three times daily for three days and then 250 mg for three more days. The patient showed improvement in her mentation after a day of thiamine therapy, confirming the diagnosis of Wernicke’s encephalopathy, although ophthalmoplegia persisted, which improved slowly within the next week.

The patient had a history of bipolar disorder, and it was later found out that she had been on lithium for many years, but in the recent past, she stopped taking the medication and was not following up with any doctor, which led to decompensation and worsening. With improvement in her mental status, a psychiatric evaluation was performed, which confirmed bipolar disorder in her with severe depression. Wernicke encephalopathy (WE) is most commonly related to alcohol abuse leading to thiamine deficiency. This strong association can lead to a higher risk of WE being overlooked when it presents in patients with other chronic medical conditions. There is documented evidence of its association with other chronic medical problems that lead to nutritional deficiencies, such as malignancy, chronic kidney disease on hemodialysis, starvation due to underlying psychiatric conditions, or hyperemesis gravidarum during pregnancy [5,6]. Although alcohol abuse cannot be completely excluded from the case, the signs that the patient had no macrocytosis, normal liver enzymes, electrolyte abnormalities such as hypomagnesemia, and a normal blood ethanol level on admission suggested against recent alcohol abuse. Also, the family who visited her house found no signs of alcohol in the apartment and reported that it looked like the patient had not left the apartment in a while and had nothing to eat at home; they saw some rotten old food. This raised a question about the patient's abnormal mental state and decompensated bipolar disorder, which made her starve chronically, leading to malnourishment, generalized weakness, and nutritional deficiencies.

Thiamine plays an important role in the Krebs cycle and the pentose phosphate cycle as a cofactor, and its insufficiency can lead to impaired glucose metabolism and glutamate metabolism and ultimately affect the production of neurotransmitters and radical scavengers [8]. Some parts of the brain, such as the cerebellar vermis and mesencephalon structures, are dependent on oxidative metabolism, and thiamine deficiency can lead to their necrosis. The majority of pathologic lesions seen in Wernicke’s encephalopathy are present in the periventricular region of the hypothalamus, thalamus, mammillary bodies, periaqueductal gray region, superior cerebellar vermis, and the floor of the fourth ventricle [1]. These lesions can be temporary or permanent based on multiple factors. Body stores for thiamine are only sufficient for up to 18 days; therefore, Wernicke’s encephalopathy can present in both acute and chronic statuses [9].

It is easier to comprehend how psychiatric disorders such as bipolar disorder can lead to nutritional deficiencies because these patients, especially when living alone, have very minimal self-care, a loss of appetite, poor dietary habits, and an increased prevalence of alcohol abuse. But to suspect the presence of Wernicke in non-alcoholic patients with psychiatric disorders and to differentiate behavioral symptoms from delirium and encephalopathy is difficult and requires clinical expertise to suspect Wernicke's encephalopathy in them.

## Conclusions

Wernicke encephalopathy is a neurological condition that can become irreversible and has a high mortality rate if intervention is not done early in the disease course. The instinctive association of Wernicke’s encephalopathy with alcohol abuse has led to multiple cases going undiagnosed. A high degree of clinical suspicion is needed in patients with psychiatric disorders who present with altered mental status to differentiate Wernicke encephalopathy from psychotic features and start thiamine early on to reduce morbidities.
